# Biomechanical Adaptations and Performance Indicators in Short Trail Running

**DOI:** 10.3389/fphys.2019.00506

**Published:** 2019-04-30

**Authors:** Glenn Björklund, Mikael Swarén, Dennis-Peter Born, Thomas Stöggl

**Affiliations:** ^1^Department of Health Sciences, Swedish Winter Sports Research Centre, Mid Sweden University, Östersund, Sweden; ^2^The Swedish Sports Confederation, Stockholm, Sweden; ^3^Royal Institute of Technology, Stockholm, Sweden; ^4^Department for Elite Sport, Swiss Federal Institute of Sport, Magglingen, Switzerland; ^5^Department of Sport and Exercise Science, University of Salzburg, Salzburg, Austria

**Keywords:** downhill running, foot forces, ground contact time, pacing, stride frequency

## Abstract

Our aims were to measure anthropometric and oxygen uptake ($\overset{˙}{V}$O_2_) variables in the laboratory, to measure kinetic and stride characteristics during a trail running time trial, and then analyse the data for correlations with trail running performance. Runners (13 men, 4 women: mean age: 29 ± 5 years; stature: 179.5 ± 0.8 cm; body mass: 69.1 ± 7.4 kg) performed laboratory tests to determine $\overset{˙}{V}$O_2 max_, running economy (RE), and anthropometric characteristics. On a separate day they performed an outdoor trail running time trial (two 3.5 km laps, total climb: 486 m) while we collected kinetic and time data. Comparing lap 2 with lap 1 (19:40 ± 1:57 min vs. 21:08 ± 2:09 min, *P* < 0.001), runners lost most time on the uphill sections and least on technical downhills (-2.5 ± 9.1 s). Inter-individual performance varied most for the downhills (CV > 25%) and least on flat terrain (CV < 10%). Overall stride cycle and ground contact time (GCT) were shorter in downhill than uphill sections (0.64 ± 0.03 vs. 0.84 ± 0.09 s; 0.26 ± 0.03 vs. 0.46 ± 0.90 s, both *P* < 0.001). Force impulse was greatest on uphill (248 ± 46 vs. 175 ± 24 Ns, *P* < 0.001) and related to GCT (*r* = 0.904, *P* < 0.001). Peak force was greater during downhill than during uphill running (1106 ± 135 vs. 959 ± 104 N, *P* < 0.01). Performance was related to absolute and relative $\overset{˙}{V}$O_2 max_ (*P* < 0.01), vertical uphill treadmill speed (*P* < 0.001) and fat percent (*P* < 0.01). Running uphill involved the greatest impulse per step due to longer GCT while downhill running generated the highest peak forces. $\overset{˙}{V}$O_2 max_, vertical running speed and fat percent are important predictors for trail running performance. Performance between runners varied the most on downhills throughout the course, while pacing resembled a reversed J pattern. Future studies should focus on longer competition distances to verify these findings and with application of measures of 3D kinematics.

## Introduction

Trail running is challenging, due to varying surfaces and inclines compared to track and road running. While the key performance indicators for running on flat, smooth surfaces are widely known, i.e., high maximal oxygen uptake ($\overset{˙}{V}$O_2 max_), lactate threshold and running economy (RE) (Morgan et al., 1989; Joyner, 1991; Jones, 1998; Midgley et al., 2006), the key factors are not clear for trail terrain. Although the effects of variations in inclines on biomechanical and physiological responses have been studied (Vernillo et al., 2017), the associations between these variables and performance is lacking. Recently, Ehrström et al. (2018) showed that relative $\overset{˙}{V}$O_2 max_ and a fatigue index using isokinetic leg exercises were positively associated to performance in shorter trail running while RE, measured on a level surface, was not with.

Trail running usually involves challenging and physically demanding uphill running, where runners need to overcome gravity to elevate their body mass as quickly and efficiently as possible. Not surprisingly, relative $\overset{˙}{V}$O_2 max_ is shown to be an important parameter for uphill treadmill and outdoor running (Staab et al., 1992; Townshend et al., 2010), as it expresses the upper limit for aerobic power in relation to body mass. However, it is noteworthy that $\overset{˙}{V}$O_2 max_ is of lesser importance for downhill running performance, as shown in outdoor running performance (Townshend et al., 2010) and laboratory-based time trials (Liefeldt et al., 1992; Staab et al., 1992; Minetti et al., 2002; Toyomura et al., 2018). Interestingly, the negative slope were not steep in two of these studies (max -11.7% outdoor and -5% indoors), but these gradients were still sufficient to reduce the importance of $\overset{˙}{V}$O_2 max_ for a fast downhill run. In addition, in a recent study of trail running on a more technical track with steeper downhill gradients none of the runners reached their $\overset{˙}{V}$O_2 max_ despite running at maximum effort (Born et al., 2017). However, for running over undulating rough terrain with great variations in gradient, the time spent descending is the strongest predictor for running performance (Kay, 2014).

To increase running speed in level terrain, runners typically increase stride length more than stride frequency (Cavanagh and Kram, 1989). In trail running, however, the challenge is multifaceted. Runners need to adapt their stride frequency and length with respect to various types of terrain and slope gradients (Giandolini et al., 2015). In a laboratory setting at -3 degrees (Liefeldt et al., 1992), and on an outdoor time-trial over an undulating course with downhill sections of -1.5 to -11.7% (Townshend et al., 2010), an increase in speed during downhill running was associated with longer stride length. Although the runners in the Townshend et al. (2010) study ran outdoors on compacted dirt and concrete footpaths, which are less technical than those in trail running, which challenges foot placement and control (Townshend et al., 2010).

For treadmill running at a constant speed representing 70% of $\overset{˙}{V}$O_2 max_ at 0% incline, increasing incline leads to increases in the ground reaction force while stride length decreases, going from 0% to uphill slopes of 2 and 7% (Padulo et al., 2013). Interestingly, in a similar study that also used a fixed velocity between different inclinations, the peak normal force during the active phase remained unchanged between uphill, level, and downhill running (Gottschall and Kram, 2005). On the other hand, in the same study by Gottschall and Kram (2005) the propulsive force impulse decreased successively from uphill, to level and downhill running.

However, changes in speed seem to play an important role for maximal normal forces as these increase with faster running on level terrain (Fourchet et al., 2012). Although faster speeds are accomplished with shorter ground contact times (GCT) in road running (Hasegawa et al., 2007), level, uphill, and downhill treadmill running at a constant speed demonstrated little or no effect on GCT (Gottschall and Kram, 2005; Vernillo et al., 2015). Interestingly, trail running seems to induce longer contact times, as shown by an increase in contact times post-trail run vs. pre-trail run using self-selected speeds (Lazzer et al., 2015). However, transferring conclusions from running on treadmills or outdoors on smooth surfaces to trail running should only be done with caution, as trail running typically includes much steeper and more technical slopes. Although studies have been conducted in conjunction with challenging trail running courses and analyzed kinematics using accelerometers and EMG (Giandolini et al., 2017), to our knowledge the kinetics of trail running has not yet been within the scope of any study.

The aims of the current study were thus to (a) analyze kinetics and stride cycle characteristics during a trail running time trial and (b) investigate physiological and anthropometric characteristics in the laboratory and relate them to trail running time trial performance in an ecologically valid environment. We hypothesized that the peak foot forces would be greater during downhill running than for level and uphill running, whereas the force impulse per step would decrease during steeper downhill running due to the shorter GCT induced by faster running speeds. Our second hypothesis was that steep downhill running is decisive for trail running performance, due to the increased difficulty of maintaining a fast running speed with enough balance and control to prevent falls.

## Materials and Methods

### Participants

This study is part of a larger study that includes a more detailed description of the physiological response to a simulated trail running competition, as already shown in the study by Born et al. (2017). The regional ethical board in Umeå, Sweden (#2014-171-31M) preapproved the research techniques and experimental protocol and the study was conducted in accordance with the Declaration of Helsinki. Participants were fully informed of the nature of the study through written and verbal information before providing written consent to participate. The runners’ characteristics are presented in Table 1.

**Table 1:** Characteristics of the subjects (mean ± SD).

Variables	Females (*n* = 4)	Males (*n* = 13)
Age (years)	30 ± 8	29 ± 4
Height (cm)	170 ± 7	183 ± 5
Body mass (kg)	59.9 ± 4.8	71.9 ± 5.6
Body fat (%)	20.0 ± 4.9	12.4 ± 3.0
Lean mass total (kg)	46.2 ± 2.6	60.9 ± 5.7
Lean mass legs (kg)	15.5 ± 2.0	20.1 ± 1.9
VO_2max_ (L ⋅ min^-1^)	3.26 ± 0.14	4.90 ± 0.64
VO_2max_ (mL ⋅ kg^-1^ ⋅ min^-1^)	55.1 ± 6.1	68.1 ± 5.8
Vertical speed (m ⋅ s^-1^)	0.31 ± 0.09	0.42 ± 0.04
RE (mL ⋅ kg^-1^ ⋅ km^-1^)	209 ± 6	210 ± 15
RE (J ⋅ kg^-1^ ⋅ m^-1^)	4.37 ± 0.05	4.31 ± 0.42


*VO_*2max*_, maximal oxygen uptake; vertical speed, vertical speed calculated from the VO_*2max*_ test; RE, running economy expressed as oxygen cost (mL ⋅ kg^-*1*^ ⋅ km^-*1*^) and energy cost (J ⋅ kg^-*1*^ ⋅ m^-*1*^).*

### General Design

**FIGURE 1:**
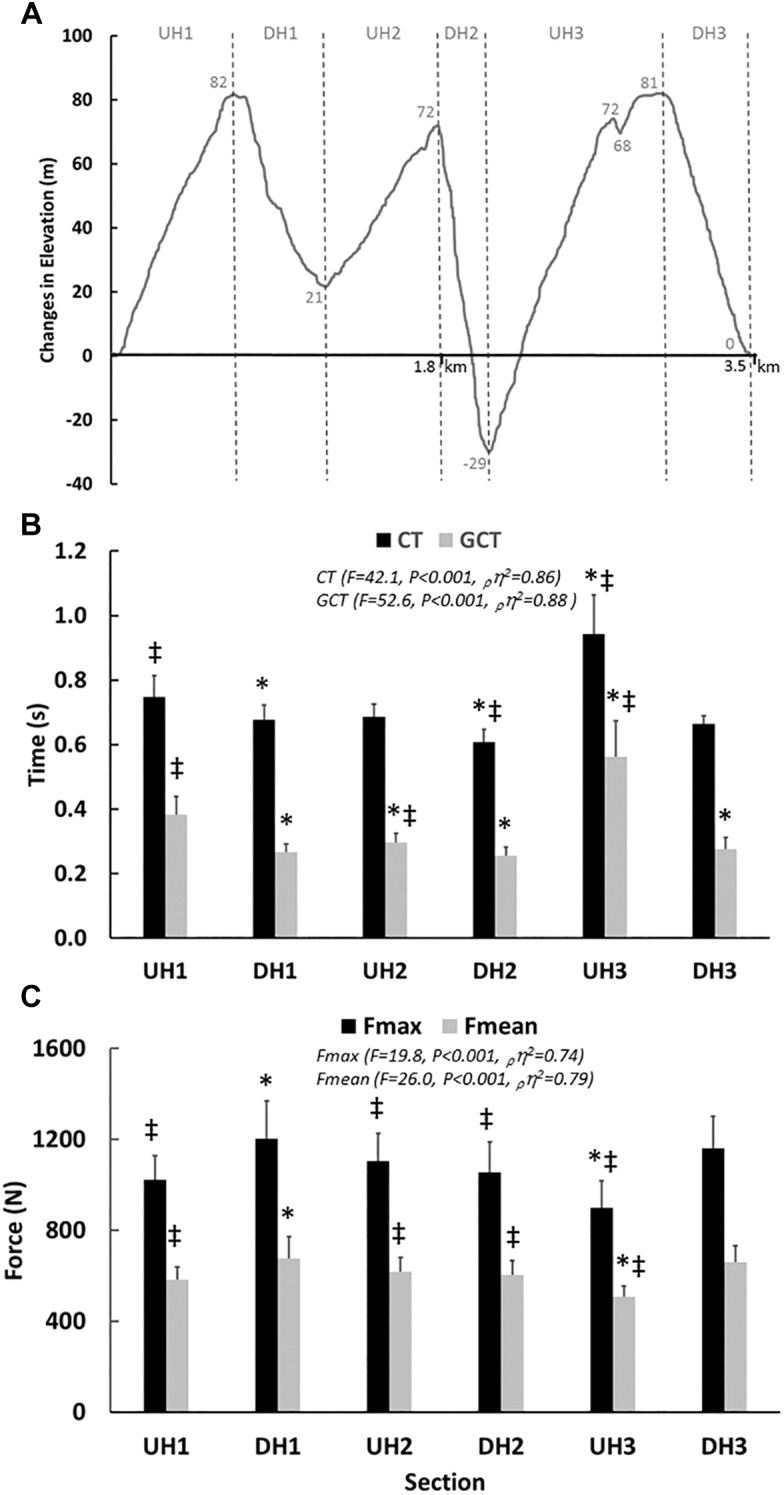
**(A)** Schematic illustration of the trail course and the specific uphill (UH) and downhill (DH) sections. **(B)** Cycle time (CT) and Ground Contact Time (GCT) expressed in s, at various sections of the course illustrated through black and gray bars, respectively. **(C)** Foot forces (N) at various sections of the course expressed as force max (F_max_) and mean (F_mean_) illustrated using black and gray bars, respectively. The *F*-, *P*-values, effect size (_p_η^2^) and power values obtained with the one way ANOVA (time sections) are presented. ^‡^*P* < 0.05 in comparison to DH1 and ^∗^*P* < 0.05 in comparison to UH1. The values given are mean ± SD.

Seventeen runners (13 men and 4 women) took part in the study. They had personal road running 10 k best times of sub 34 min for the men (range 29–33 min) and 38 min for the women (range 34–38 min). In addition, several runners had podium placings in elite level international trail running competitions. All participants underwent anthropometric and physiological laboratory tests as well as an outdoor trail run within the same week. Anthropometric and physiological laboratory tests were conducted (lean mass, fat percent, $\overset{˙}{V}$O_2 max_, RE) on day 1, while on day 2 participants performed two laps solo run on a 3,524 m trail running course (simulated running competition) (Figure 1A). The track consisted of three major uphill (UH) and downhill (DH) sections, with a total elevation gain of 243 m for each lap. More specifically, the third and last UH of the course consisted of a gradually inclined gravel path and the third DH section started with a steep rocky section. The course imitated the typical conditions for a trail run competition, including a mix of mud, gravel and dirt with technical ascents and descents. Before the field time trial, all participants ran the outdoor trail course to diminish the potential effect of learning on performance (Easthope et al., 2014). The runners applied their usual pre-competition routine and inspected the most crucial parts of the trail course at their own pace the day before. All runners refrained from strenuous exercise and alcohol for 24 h before both the laboratory tests and field time trial.

### Anthropometrics

Participants arrived in the early morning in a fasting state, after not consuming food or drink for at least 8 h. Their height and weight were measured on a calibrated scale (7014 SECA 764, Benson Avenue, CA, United States) in minimal clothing. To determine body composition, the runners were scanned with a whole-body dual-energy x-ray absorptiometry (iDXA) (Encore 2011, Version 13.60, General Electric Company, Madison, WI, United States). Calibration of the iDXA was performed using a phantom model the same day as the tests (Quality Control, QC). The coefficient of variation (CV) was below 0.20% for all QC performed during the study.

### Oxygen Uptake

$\overset{˙}{V}$O_2 max_ and running economy (RE) were measured with a portable metabolic cart, MetaMax3B_R2 (Cortex Biophysik GmbH, Leipzig, Germany). The athletes were fitted with a correctly sized mask that covered the mouth and nose (7450 Series V2^TM^ Mask, Hans Rudolph Inc., Shawnee, United States). Before all tests, the gas analyzer’s oxygen (O_2_) and carbon dioxide (CO_2_) sensors were calibrated using a two-calibration procedure with ambient air conditions (20.93% O_2_ and 0.03% CO_2_) and the anticipated expiratory gas percent using calibration gas containing 15% O_2_ and 5% CO_2_ (UN 1950 Aerosols, Cortex Biophysik GmbH, Leipzig, Germany). The flow volume was calibrated using a 3-L syringe (M9474-C, Medikro Oy, Kuopio, Finland). Before the study, the metabolic cart was validated using a metabolic simulator (Model 17057, Vacumed, Ventura, United States). For details see the previous paper from our group (Born et al., 2017).

### Running Economy and $\overset{˙}{V}$O_2 max_

Oxygen and energy cost as an indicator of RE were calculated using a treadmill (RL3000, Rodby, Innovation AB, Vänge, Sweden) set at a submaximal workload that was predetermined at 14 and 16 km ⋅ h^-1^, respectively, for women and men, at a gradient of 1° for 5 min. The chosen speeds were substantially lower than their personal best 10 km times and represented < 1.0 in respiratory exchange ratio (RER). RE were expressed as oxygen cost and energy cost (E*_cost_*), expressed as mL ⋅ kg^-1^ ⋅ km^-1^, and J ⋅ kg^-1^ ⋅ m^-1^, respectively. The calculation of oxygen cost was determined using $\overset{˙}{V}$O_2_ expressed as mL ⋅ kg^-1^ ⋅ min^-1^ and running pace expressed as min ⋅ km^-1^:

$$\text{Oxygen cost=}\overset{˙}{V}O_{2}\cdot \text{running pace}$$

The energy expenditure *E_exp_* (kcal ⋅ min^-1^) was calculated using the Weir equation (Weir, 1949):

$$E_{\exp}\text{=}((1.1\cdot RER)+3.9)\cdot \overset{˙}{V}O_{2}$$

where RER is the respiratory exchange ratio and $\overset{˙}{V}$O_2_ is expressed as L ⋅ min^-1^. To calculate the *E_cost_*, the unit of work of *E_exp_* was transformed from kcal to J, and divided by body mass (m) and running speed (v) expressed as m ⋅ min^-1^:

$$E_{\cos t}\text{=}\left(4186\cdot E_{\exp}\cdot m^{-1}\right)\cdot v^{-1}$$

The $\overset{˙}{V}$O_2 max_ test was performed in association with the RE test, following a 10 min break. Participants used the same speed as in the RE test, while the inclination increased by 1° per minute until volitional fatigue. At least two out of three criteria needed to be reached for a valid maximal effort: (1) a leveling off in $\overset{˙}{V}$O_2_ defined as an increase <150 mL ⋅ min^-1^; (2) RER > 1.10; and (3) rating of perceived exertion ≥ 18 for sensation in both legs and breathing on the Borg 6–20 scale (Borg, 1970).

### Vertical Velocity

The vertical velocity (m ⋅ s^-1^) was calculated during the $\overset{˙}{V}$O_2 max_ test as:

$$v_{vert}=v\cdot \sin\alpha$$

where *v* is the speed of the belt on the treadmill and α is the incline of the treadmill in degrees. To calculate the maximal incline achieved by the runner, the incline of the last completed stage and the time the runner stayed at the consecutive incline were used accordingly:

$$\text{Maximal incline=}{workload}_{i}+\left(t÷60\right)$$

Workload*_i_* is the last completed stage in degrees and *t* is the time the runner lasted on the final uncompleted workload.

### Biomechanical Methods

Kinetic data were collected with the OpenGo system (Moticon GmbH, Munich, Germany), consisting of two pressure insoles (one in each shoe, each containing 13 capacitive sensors) that measured plantar pressure and 3D accelerations at 50 Hz. The plantar pressure data were used to compute plantar normal forces for each foot. Each sensor insole incorporates a processing unit, memory (16 MB flash memory each) and a wireless module for data transmission and control of the sensor insole. No external devices or cables were needed to operate the system. The OpenGo sensor insoles were factory calibrated with homogeneously distributed loads, covering the specified load range from 0 to 40 N/cm^2^. Based on the specifications from Moticon (Stöggl and Martiner, 2017), no further calibration is needed within the specified lifetime of 100 km of running; therefore, no supplementary calibration was performed for the purpose of the present study. However, the pressure data was zeroed prior to each time trial. Participants wore their individual running shoes with the original insole removed and replaced with the OpenGo sensor insoles.

Section times were established using a wireless timing gate system (iMicroGate, Bolzano, Italy) positioned along the course. These specific points of interest were all well marked and the runners had identified them during the familiarization runs. Furthermore, manually added timestamps at these points of interest were used for identification and to ensure that the kinetic analyses were performed at the same positions along the track for each runner. To study whether there were changes during the most technical DH and UH sections, the second DH (DH2), third UH (UH3), and third DH (DH3) section were further divided into three subsections: start, middle and end based on distance (m) of the sections. The sensor insoles and data logger were time synchronized at the beginning of each trial by performing a distinct stamp with one leg while the time stamp button on the data logger was pressed simultaneously.

### Spatiotemporal Variables

Twenty consecutive running (walking) strides were analyzed within each preselected section of the course. These sections were based on the global positioning system (GPS) and barometric data, collected by the portable metabolic cart. For kinetics, the impulse of force, peak force and mean force during stance were calculated. Kinematic data, such as cycle time (CT), GCT and swing time, were automatically determined by combining the force data with the internal accelerometer data. To do this, a force threshold was first applied to the total force value to roughly detect heel-strike and toe-off during running and in some cases walking (e.g., very steep UH). Within a window of 100 ms around these time points, the algorithm used the local minima in the sequence of unfiltered acceleration values in Y-direction (anterior-posterior) to fine-tune the detection time points (Stöggl and Martiner, 2017). Processing of the data was managed using IKE-master software (IKE-Software Solutions, Salzburg, Austria) and MS Excel 2010 (Microsoft Corporation, Redmond, WA, United States).

### Statistical Analyses

The relationships between trail running race performance (i.e., race time), and laboratory and field biomechanical data were evaluated with Pearson’s product moment correlation coefficients. Partial correlation assessed the relationships between anthropometric data and race performance (time), controlling for confounding variables expressed as *r*_,A,B | C_ when A and B are correlated and control for the effect of C. To compare laboratory data between sexes, an independent student’s *t*-test was used. A two-way repeated ANCOVA was performed to compare different time sections between the two laps, where the covariate was set as sex. A step-wise multiple linear regression was used to predict the importance of different section times on overall performance. Pressure insole data was evaluated using one-way repeated ANOVA. For the ANCOVA and ANOVA, a Bonferroni *post hoc* test was also used if there was a significant global difference. The Greenhouse-Geisser correction was used if the sphericity was violated. Effect size for variance analysis was partial eta square and Hedge’s *g* for paired analysis using a weighted pooled standard deviation. The meaningfulness of the results was ranked as low (0.2–0.49), medium (0.5–0.79) and large (>0.8) (Thomas et al., 1991). All statistical analysis were performed with SPSS (IBM Corp. Released 2017. IBM SPSS Statistics for Windows, Version 25.0. Armonk, NY: IBM Corp). For statistical significance, the α value was set *a priori* at < 0.05. Results are expressed as mean values ± standard deviations (SD).

## Results

### General Performance Data

**FIGURE 2:**
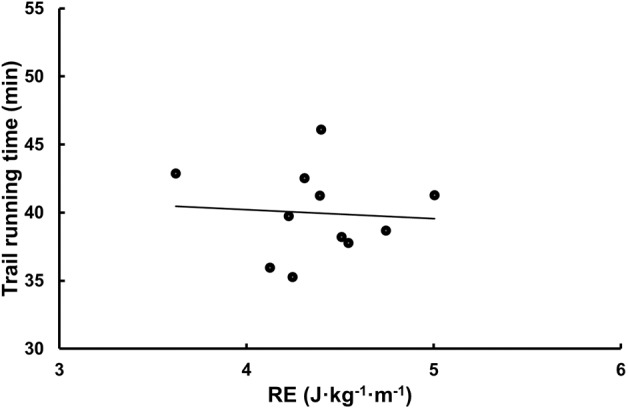
The relationship between RE (J ⋅ kg^-1^ ⋅ m^-1^) and trail running time (min).

The mean total time for completing the whole trail running course was 40:48 ± 4:04 min. Laboratory data associated with performance were $\overset{˙}{V}$O_2 max_ (*r* = -0.71, *P* = 0.005 and -0.82, *P* < 0.001 for L ⋅ min^-1^ and mL ⋅ kg^-1^ ⋅ min^-1^) and vertical uphill speed (*r* = -0.85, *P* < 0.001), while RE expressed as mL ⋅ kg^-1^ ⋅ km^-1^ (*r* = -0.16, *P* = 0.60) or J ⋅ kg^-1^ ⋅ m^-1^ (*r* = -0.07, *P* = 0.83) were not (Figure 2). Total race time was related to lean mass (*r* = -0.63, *P* = 0.015) and fat percent when controlling for total lean mass (*r*_totaltime,fatpercent | totalleanmass_ = 0.73, *P* = 0.005) but not mean mass of the legs (*r*_totaltime,leanmasslegs | totalleanmass_ = 0.42, *P* = 0.15).

### Performance in Various Sections

The runners’ intra-individual performance showed performance decreases of 88 ± 37 s from the first to the second lap (19:40 ± 1:57 min vs. 21:08 ± 2:09 min, *P* < 0.001, CI = -109 to -67 s, *g* = 0.71). The sectional time loss between the two laps depended on the terrain (*P* < 0.001, _ρ_η^2^ = 0.614) with the greatest time loss on the three UH sections, followed by DH2 and DH1 (*P* < 0.001, Table 2). There was no change in the last DH (DH3) between lap 1 and 2. There was no inter-difference between the three UH sections with respect to time delta changes between lap 1 and 2 (UH1 = 29.0 ± 18.5 s, UH2 = 18.9 ± 7.5 s, UH3 = 19.7 ± 14.2 s, *P* > 0.05, Table 2). The UH sections constituted 54.4 and 56.0% of the total time for lap 1 and 2, respectively. The performance between runners varied the most during the DH sections explained by the greater CV (Table 2). The largest CV was for the most technical DH part (DH3); showing values greater than 20% in CV on both the first and second laps. The stepwise multiple regression model showed that these sections were related to overall performance in the following order UH2 (*P* < 0.001), DH2 (*P* = 0.009), and UH1 (*P* = 0.018) with an adjusted *R*^2^ of 0.976. The prediction model was =

Overall running time=44.251+(6.42⋅UH2)+(6.00⋅DH2)+(2.44⋅UH1)

**Table 2:** Times (s) for the different sections from lap 1 and 2.

	UH1	DH1	UH2	DH2	UH3	UH3	DH3	DH3
						(flatter)	(rocks)	(middle, end)
Lap 1 (s)	205 ± 20	129 ± 15	211 ± 23	92 ± 13	246 ± 31	25 ± 2	12 ± 4	116 ± 16
CV	10	11	11	14	12	9	33	14
Lap 2 (s)	233 ± 24^†^ 10	135 ± 14^∗^ 11	230 ± 26^†^ 11	100 ± 16^†^ 16	266 ± 34 13	26 ± 3 11	12 ± 3 25	114 ± 13 11
CV	10	11	11	16	13	11	25	11


*Data is expressed as means ± SD and CV%. Times for the different sections are presented as mean values ± SD in seconds (s). UH, Uphill; DH, Downhill. Coefficient of variation (CV) is presented as a percentage. The results are based on n = 14 out of the total number of participants (n = 17), due to missing sections times for three runners. Difference from lap 1 ^∗^P < 0.05 and ^†^P < 0.001.*

### General Pressure Insole Data

Based on the challenging terrain and demanding outdoor conditions on some of the test days, the pressure insole data could be analyzed for only nine runners. The overall cycle time during the UH sections was, in total, longer than for the DH sections (0.84 ± 0.09 s vs. 0.64 ± 0.03 s, *P* < 0.001, *g* = 3.07, Figure 1B). Further, GCT generally increased on UH sections to almost double that of the DH sections (0.46 ± 0.9 s vs. 0.26 ± 0.03 s, *P* < 0.001, *g* = 2.99, Figure 1B). Increased force impulse was shown on the UH compared to the DH sections (248 ± 46 Ns vs. 175 ± 24 Ns, *P* < 0.001, *g* = 1.99, Tables 3, 4). This increase in force impulse during UH running (walking) was related to the GCT (*r* = 0.904, *P* < 0.001) and not the mean (*r* = 0.287, *P* = 0.454) or peak foot forces (*r* = 0.277, *P* = 0.470). Comparing the UH to the DH sections, both peak (959 ± 104 N vs. 1106 ± 135 N, *P* < 0.01, *g* = 1.22) and mean foot forces (541 ± 46 N vs. 626 ± 75 N, *P* < 0.001, g = 1.37) were lower for the UH sections (Figure 1C and Table 4).

**Table 3:** Stride frequency and impulse on different sections.

	UH1	DH1	UH2	DH2	UH3	DH3	*F* = values	*P* = values	_ρ_η^2^
Stride frequency (strides ⋅ min^-1^)	81 ± 6^†^	89 ± 6^∗^	88 ± 5^∗^	100 ± 7^∗†^	66 ± 9^∗†^	91 ± 3^∗^	*F* = 61.1	*P* < 0.001	0.90
Impulse (N ⋅ s)	232 ± 46^†^	193 ± 28^∗^	194 ± 25	164 ± 19^∗^	287 ± 52^†^	192 ± 22	*F* = 24.2	*P* < 0.001	0.78


*One-way repeated ANOVA for pressure insole data measured normally. Effect size is expressed as partial eta squared (_ρ_η^*2*^). All data are expressed as group mean values ± SD.^∗^P < 0.05 different from UH1 and ^†^P < 0.05 different from DH1.*

**Table 4:** Kinetics for DH2, UH3, and DH3.

	Start	Middle	End	*P* = values, *F* = values, _ρ_η^2^
**DH2**				
Force max (N)	1108 ± 156^‡^	929 ± 107^∗†^	1097 ± 149^‡^	*F* = 21.6, *P* < 0.001, _ρ_η^2^ = 0.73
Force mean (N)	635 ± 84^‡^	523 ± 53^∗†^	626 ± 74^‡^	*F* = 22.6, *P* < 0.001, _ρ_η^2^ = 0.74
Impulse (N ⋅ s)	169 ± 8	145 ± 9	170 ± 7	*F* = 6.0, *P* = 0.011, _ρ_η^2^ = 0.43
**UH3**				
Force max (N)	935 ± 138	843 ± 121	917 ± 148	*F* = 2.9, *P* = 0.09, _ρ_η^2^ = 0.29
Force mean (N)	534 ± 68	481 ± 50	509 ± 64	*F* = 2.4, *P* = 0.127, _ρ_η^2^ = 0.26
Impulse (N ⋅ s)	272 ± 65	311 ± 99	277 ± 56	*F* = 0.8, *P* = 0.466, _ρ_η^2^ = 0.10
**DH3**				
Force max (N)	1143 ± 162	1159 ± 139	1180 ± 151	*F* = 0.6, *P* = 0.494, _ρ_η^2^ = 0.08
Force mean (N)	648 ± 86	657 ± 76	673 ± 80	*F* = 0.7, *P* = 0.515, _ρ_η^2^ = 0.09
Impulse (N ⋅ s)	203 ± 37	186 ± 21	186 ± 21	*F* = 2.3, *P* = 0.167, _ρ_η^2^ = 0.25


*One-way repeated ANOVA for pressure insole data. Effect size is expressed as partial eta squared (_ρ_η^*2*^). All data are expressed as group mean values ± SD. Bonferroni post hoc analysis. ^∗^Difference from Start; ^‡^Middle; ^†^End.*

### Spatiotemporal Data in Various Sections

Stride frequency and GCT were strongly related on the UH sections (UH1: *r* = -0.936, *P* < 0.001; UH3: start *r* = -0.986, *P* < 0.001; UH3: middle *r* = -0.939, *P* = 0.001; UH3: end *r* = -0.959, *P* < 0.001). Swing time was negatively associated with stride frequency during DH1 (*r* = -0.798, *P* = 0.01) and DH2 (start *r* = -0.865, *P* = 0.003; middle *r* = -0.761, *P* = 0.017; end *r* = -0.856, *P* = 0.003). Force impulse was associated with GCT for all UH sections (UH1: *r* = 0.852, *P* = 0.004; UH2: *r* = 0.763, *P* = 0.017, UH3: start *r* = 0.949, *P* < 0.001; middle *r* = 0.948, *P* < 0.001; end *r* = 0.933, *P* = 0.001). During the DH sections there was an association between stride frequency and GCT on the steepest section (>30% inclination) and the most technical part, which consisted of rocks (DH2 middle *r* = -0.801, *P* = 0.009 and DH3 rocks *r* = -0.711, *P* = 0.048).

### Subsections of the Downhill and Uphill Sections

Stride frequency changed on various sections, subdivided into start, middle and end during the DH2 (start = 91 ± 6 strides ⋅ min^-1^, middle = 113 ± 12 strides ⋅ min^-1^ and end 96 ± 7 strides ⋅ min^-1^, *F* = 22.0, *P* < 0.001, _ρ_η^2^ = 0.73) with an increase in the middle subsection compared to start and end (*P* = 0.001 and *P* = 0.011, respectively). During the steep UH3 there was no change in stride frequency between the different subsections (start = 72 ± 14 strides ⋅ min^-1^, middle = 60 ± 10 strides ⋅ min^-1^ and end 65 ± 11 strides ⋅ min^-1^, *F* = 3.1, *P* = 0.076, _ρ_η^2^ = 0.31). On DH3, there was a global change in stride frequency for the start, middle and end (start = 86 ± 7 strides ⋅ min^-1^, middle = 92 ± 3 strides ⋅ min^-1^, end = 95 ± 5 strides ⋅ min^-1^, *F* = 7.2, *P* < 0.01, _ρ_η^2^ = 0.51).

On the UH compared to the DH sections, the GCT was prolonged (*P* < 0.001). Within single sections it remained unchanged for DH2 (start = 0.25 ± 0.02 s, middle = 0.26 ± 0.04 s, end = 0.25 ± 0.02 s, *F* = 0.8, *P* = 0.461, _ρ_η^2^ = 0.09), DH3 (start = 0.30 s ± 0.08 s, middle = 0.27 ± 0.04 s, end = 0.26 ± 0.03 s, *F* = 2.0, *P* = 0.201, _ρ_η^2^ = 0.22) and UH3 (start = 0.51 ± 0.16 s, middle = 0.63 ± 0.17 s, end = 0.55 ± 0.15 s, *F* = 1.8, *P* = 0.204, _ρ_η^2^ = 0.20).

## Discussion

The major findings of the study show that the intra-individual time lost between lap 1 and 2 was greater for the UH sections, while the largest variation between runners was on the DH sections. From a biomechanical perspective the stride frequency showed large variation with the slowest stride frequency in the UH sections, and stride frequency almost doubled on the steep DH sections. In addition, stride frequency was negatively associated with GCT, especially during UH. The highest forces are displayed on the DH sections, whereas the force impulse is greater on the UH sections, which is related to the prolonged GCT. Absolute and relative values in$\overset{˙}{V}$O_2 max_, as well as anthropometric variables such as body fat percentage were related to performance, while RE measured on level terrain in the laboratory seems irrelevant.

### Performance

The runners were encouraged to complete the trail course as fast as possible under the form of a time trial exercise. The general pattern of run pace for all runners was a faster first lap than the second lap (positive pacing). When analyzing the single sections, faster lap 1 performance was only seen on the first four sections of the two laps (first two UH and DH sections), with even match times for the last part of UH, which was a gravel section, and the beginning of last DH that was a technical rocky section. Although the runners probably experienced high levels of physical exertion on these sections, on the last tough UH and consecutive flatter end of the UH3 section, the runners had no decline in performance between laps. The only section that was faster on the second lap was the last DH section. Altogether, this type of pacing is defined as a reversed J-shaped strategy with a slow-down in the middle to excel in the latter part (Abbiss and Laursen, 2008). Previously it has been shown that pacing in top class cross-country runners is different when compared with less successful runners. Less successful runners demonstrated positive pacing while the best runners used even pacing (Esteve-Lanao et al., 2014). In the current study, athletes generally showed a J-shaped pacing pattern. Furthermore, the most significant sections for individual performance, i.e., time to complete the course, were by the least technical UH sections and the steepest DH sections. This is an interesting perspective, as it gives the insight that high-level roadrunners with less specific trail running skills but with a high relative $\overset{˙}{V}$O_2 max_ could perform well on less challenging courses. However, as the steepest part of the DH sections requires excellent descending skills, elite trail runners have the potential to make up time with less effort due to the low oxygen demand for a given downhill running speed. However future studies need to prove this potential.

### Uphill and Downhill Sections

Although there were two UH sections that impacted overall performance, the most significant for the overall outcome was the least technical part (UH2), with a mostly even gravel surface. Therefore, it seems that other features must be considered for inclines, such as aerobic power and, probably, an efficient level terrain running technique where RE has been interrelated to modestly steep uphill’s (Breiner et al., 2018). Moreover, the anaerobic contribution could also influence the speed for this group of runners with a similar $\overset{˙}{V}$O_2 max_, which has been demonstrated in endurance athletes with similar aerobic power and different performance levels (Lucia et al., 1998). Although a short contact time is a result of a faster running pace and not vice versa, the contact time during the UH2 section was the shortest for uphill running and closer to road running contact times (Hasegawa et al., 2007). Accordingly, the cycle time was longer overall for UH compared to DH running, while the differences in relative contact time were proportionally even greater, i.e., a longer duty cycle with less aerial time in proportion to the complete stride cycle for UH than DH running. Additionally, on DH2 the contact time remained constant although the stride frequency varied, which can be only achieved with a shorter swing phase.

The importance of skilled DH running is apparent in two ways. The most technical, but rather short, section of uneven rocks had a very high CV within the group (∼30%). This within-group variation is similar in many ways to the conclusions of Kay (2014), who showed a large standard deviation in times to complete difficult descents between runners and was related to overall performance. Another key factor in performance is the skill of increasing speed during very steep DH running, as shown in the multiple regression analysis. Furthermore, when descending such a steep DH section, $\overset{˙}{V}$O_2 max_ is of minor importance as it is not attainable in this type of terrain (Staab et al., 1992; Townshend et al., 2010). Nevertheless, it seems that the key factor is to increase stride frequency and to be able to switch between different stride frequencies, as displayed within the subdivided sections of the descent. In addition, as pointed out in the study by Giandolini et al. (2017), a variation in stride pattern might reduce neuromuscular fatigue subsequently. In our study, the stride pattern variation might have influenced UH running capacity on the subsequent sections. In great contrast to running on undulating terrain on smooth surfaces, where the stride frequency remains constant (Townshend et al., 2010) it seems that alternating the stride pattern is necessary to optimize short trail running performance. The change in stride frequency is a prerequisite, as the terrain is constantly changing so the adjustment of only stride length seems unlikely to achieve optimal foot placement. This finding is also supported by previous data from Giandolini et al. (2015) showing an atypical foot strike pattern for a world class trail runner during an actual trail running competition.

### Foot Forces

Kinetic data from trail running is sparse and, to our knowledge, foot forces when running in such difficult terrain have not yet been presented. In a controlled environment such as treadmill running, some general patterns are key, such as an increase in braking force with increased descending at low speed (3 m ⋅ s^-1^), while impact peaks are minimized during steeper ascents (Gottschall and Kram, 2005). As shown in the current study, the lower force impulse during DH vs. UH running gives some indication that the change of momentum is greater for UH running. Interestingly, in most circumstances the need to store elastic energy is likely crucial for increasing running efficiency (Alexander, 1991). However, during steep DH running, elastic energy is likely less important since mechanical energy must be dissipated (Snyder et al., 2012). In this context, on steep DH sections, runners need to constantly lower the center of mass and reduce vertical movement of the center of mass to a minimum value. Based on the current study, it can be assumed that the strategy for enabling this maneuver is to utilize faster stride frequencies.

Runners experienced lower mean and maximal forces during UH than for DH. This confirms data from laboratory studies using pre-set velocities (Gottschall and Kram, 2005). When trail running, the maximal forces during descending increase by almost 400 N compared to ascending. While the current study used normal forces and not horizontal forces, the increase in braking forces would probably be even greater. Nevertheless, given the importance of withstanding these higher forces during DH compared to UH running, eccentric leg strength probably should be evaluated in future studies of trail runners. Although it was outside the measured variables in the current study, the foot strike pattern could influence eccentric work as a forefoot strike pattern can modulate the vertical GRF (Kuhman et al., 2016). The use of forefoot striking running during downhill running has also been suggested (Kowalski and Li, 2016). However, these studies were performed indoors and have not used such steep descents or surfaces characterizing trail running.

### Laboratory Data

Absolute and relative values of $\overset{˙}{V}$O_2 max_ were strongly association to performance during the trail run. Furthermore, relative $\overset{˙}{V}$O_2 max_ correlated slightly more strongly than the absolute values. While the relation to relative $\overset{˙}{V}$O_2 max_ and trail running performance has recently been demonstrated (Ehrström et al., 2018; Scheer et al., 2018) the importance of absolute $\overset{˙}{V}$O_2 max_has not. This provides an interesting perspective in this group of runners, as low weight *per se* is not connected to performance when running over rough undulating terrain. Possibly the strong association between both absolute and relative $\overset{˙}{V}$O_2 max_ and performance could be a question of scaling for trail running. Although $\overset{˙}{V}$O_2 max_ is confirmed to be an important performance variable in this study and others (Ehrström et al., 2018; Scheer et al., 2018) this confines most likely within trail runners. Jensen et al. (1999) showed that road runners were clearly more effected by the rough terrain, i.e., greater increase in oxygen cost, and consequently a decline in performance compared to orienteers although demonstrating similar $\overset{˙}{V}$O_2 max_ between groups. Furthermore, anthropometric characteristics showed that a low fat percentage, independent of lean mass, is critical for running on undulating terrain with steep slopes.

In the current study, there was no relation between RE determined in the laboratory, expressed as energy or oxygen consumption, and trail running performance. This contrasts with the study by Paavolainen et al. (1999), which demonstrated strong correlations between RE and running performance on level terrain (McLaughlin et al., 2010). Another interesting finding is that RE was equal for both sexes. This was very noticeable, as shown by very low effect sizes for both oxygen cost and energy cost in comparisons between women and men. Additionally, as shown in the current study, the vertical speed determined by a standardized procedure in the laboratory was related to trail running performance. While inclined uphill protocols have previously been used (Scheer et al., 2018), our study is the first to confirm the relation between trail running performance and uphill running performance in the lab. Moreover, a protocol for downhill running could be of interest in investigating the runner’s ability to withstand muscular fatigue and leg muscle soreness. Overall, laboratory data that is important for evaluating trail runners should involve relative and absolute $\overset{˙}{V}$O_2 max_, vertical running speed and anthropometric data like fat percent and lean mass.

Overall, uphill running produced the greatest impulse due to longer contact times, while downhill running generated the highest peak forces. Trail running challenges the ability to alter stride frequency, contact time, swing time and kinetics in general. Runners showed a reversed J pacing pattern while performances between runners varied the most on downhills throughout the course. The time lost between laps was greatest for the uphill sections.

Although the study was conducted with advanced technology this also limits the study in some ways. It is impossible to equip multiple runners with identical technology, as it is very costly. Consequently, the outdoor trials were performed on multiple days. The day-to-day variation in weather conditions can therefore be seen as a limitation, while the question on much this could interfere with the outcome of the performance is difficult to estimate. However, the inter-individual changes in performance are still very robust as the runners performed two consecutive laps with exactly the same prerequisites. One other limitation was the different speeds for determination of RE between men and women. However, the chosen speeds needed to be relative to the personal best 10 k personal best, but with not a substantial anaerobic contribution, i.e., respiratory exchange ration below 1.0. Additionally, we were the first one to show kinetic data on elite level female trail runners and further none of the other studies collected data during actual trail running. Even though the number of participants from whom we successfully collected kinetic data is low, it is still equal to other relevant studies in trail running (Ehrström et al., 2018; Scheer et al., 2018).

## Perspective

Interestingly, when using different slope protocols from level terrain to terrain as steeply uphill as 25%, $\overset{˙}{V}$O_2 max_ was consistent between various protocols while RE, expressed as oxygen cost per meter, increased with greater slopes (Balducci et al., 2016). This further implies that the design of the RE protocol might be vital in providing valuable insight into physiological characteristics of trail runners. Although Breiner et al. (2018) showed that $\overset{˙}{V}$O_2_ at a moderate exercise intensity on not so steep uphill or downhill running is related to level runners $\overset{˙}{V}$O_2_, the relation between energy cost on steep uphill, downhill and level running in trail runners is still missing. In addition, studying how runners alter the kinetics using specific running techniques in various terrains would be beneficial. Furthermore, at longer distances musculoskeletal problems and fatigue might very well change the kinetics and pacing. In terms of performance gains, trail runners with less experience should target to improve downhill running skills. For the more advanced trail runner, to improve uphill running skills seems to favor performance in shorter trail running competitions.

## Ethics Statement

The regional ethical board in Umeå, Sweden (#2014-171-31M) preapproved the research techniques and experimental protocol and the study was conducted in accordance with the Declaration of Helsinki. Participants were fully informed of the nature of the study through written and verbal information before providing written consent to participate.

## Author Contributions

GB and MS conceived and designed the experiments. GB, MS, D-PB, and TS performed the experiments, analyzed the data, and prepared the manuscript. All authors read and approved the final manuscript.

## Conflict of Interest Statement

The authors declare that the research was conducted in the absence of any commercial or financial relationships that could be construed as a potential conflict of interest.
